# Ventricular assist devices and non-cardiac surgery

**DOI:** 10.1186/s12871-015-0157-y

**Published:** 2015-12-19

**Authors:** S. Michael Roberts, David G. Hovord, Ramesh Kodavatiganti, Subramanian Sathishkumar

**Affiliations:** 1 Department of Anesthesiology, Penn State Milton S Hershey Medical Center, 500, University drive, 17033 Hershey, PA USA; 2 Department of Anesthesiology, University of Michigan Health System, 1500 E Medical Centre Drive, Ann Arbor, 48109 USA; 3 Department of Anesthesiology, Children`s Hospital of Philadelphia, University of Pennsylvania, Perelman School of Medicine, 34th Street and Civic Center Boulevard, Suite 12NW40, Philadelphia, PA 19104 USA

**Keywords:** Heartware, Heartmate, Ventricular assist device, Perioperative management

## Abstract

The use of ventricular assist devices has expanded significantly since their approval by the Food and Drug Administration in the United States in 1994. In addition to this, the prevalence of heart failure continues to increase. We aim to provide an overview of perioperative considerations and management of these patients for non-cardiac surgery. We performed a Medline search for the words “ventricular assist device,” “Heartmate” and “HeartWare” to gain an overview of the literature surrounding these devices, and chose studies with relevance to the stated aims of this review. Patients with ventricular assist devices are presenting more frequently for surgery not related to their cardiac pathology. As the mechanically supported population grows, general anesthesiologists will be faced with managing these patients, possibly outside of the tertiary care setting. The unique challenges of this patient population can best be addressed by a thorough understanding of ventricular assist device physiology and a multidisciplinary approach to care.

## Background

Since the approval of ventricular assist devices (VADs) by the Food and Drug Administration (FDA) in the United States in 1994, their indications and prevalence have continued to expand [1]. Thanks to advances in cardiovascular care allowing for survivability of cardiovascular insults, the prevalence of heart failure continues to increase with an estimated 670,000 new cases each year, $34 billion in associated healthcare costs annually, and a total population of 5.8 million heart failure patients in the United States [2, 3]. Initially, these support devices were approved to bridge end-stage heart failure patients awaiting transplant, termed “bridge-to-transplant”. However, approximately 30,000 patients are listed for heart transplantation per year but only 3500 are performed [4]. The findings of improved hemodynamics, end-organ function, exercise tolerance, and overall improvement in quality of life thanks to left ventricular assist device (LVAD) therapy led to device implantation for non-transplant candidates as well, termed “destination therapy”. The REMATCH and INTrEPID trials demonstrated a significant reduction in all-cause mortality and nearly tripled 2-year survival comparing LVAD implantation to optimal medical therapy [5, 6]. LVAD therapy has even been shown to transition NYHA (New York Heart Association) Class IV patients to Class I or II [7]. These patients are able to be discharged from the hospital and, thanks to their improved survivorship, may present for non-cardiac surgical procedures unrelated to their heart failure pathology. Prior studies have demonstrated that 23–27 % of LVAD patients undergo non-cardiac surgery [1]. As the mechanically supported population grows, general anesthesiologists will be faced with managing these patients more frequently, possibly outside of the high-level tertiary care setting [7, 8]. The authors intend to provide an overview of currently available LVAD technology as well as perioperative considerations and management of these patients for non-cardiac surgery.

### Review

#### Current ventricular assist devices

Since their first use in the early 1990s, VADs have developed and undergone multiple evolutions resulting in the devices currently in use. First-generation devices attempted to assist the failing ventricle by assuming its pump function. These pumps were either pneumatically or electrically driven and operated in a fill-to-empty mechanism, which produced pulsatile flow in an asynchronous fashion with the native ventricle [9]. These devices were noisy, large and required the pump chambers be extracorporeal or implanted into the abdomen with larger percutaneous drivelines [10]. These complex devices had a high incidence of complications including mechanical failure, infection and thromboembolic events [11].

Second generation devices abandoned efforts to replace the function of the ventricle and instead unload volume from the failing ventricle in a continuous, nonpulsatile fashion. An inflow cannula is placed in either the left atrium or, more commonly, the apex of the left ventricle (LV) and blood is pumped via a rotating impeller in an axial or centrifugal fashion to the ascending aorta [11, 12]. These devices are vastly smaller, totally implantable generally into the pericardial space, silent, dependable, and require less anticoagulation than most of the prior generation devices thanks to their valveless, continuous-flow systems [10–13]. The newest third-generation devices employ the same continuous-flow mechanisms, but make small improvements, such as hydrostatic or magnetic bearings which minimize shear stress and the incidence of thrombus formation [10]. Though multiple second and third generation devices are in development and undergoing investigative trials, there are currently two devices approved by the FDA for use in adults [14, 15].

**Fig. 1:**
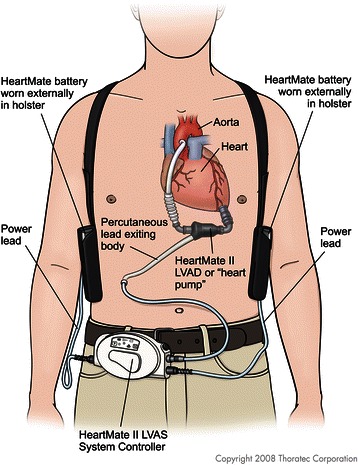
Schematic of Heartmate 2, with battery. Legend – The power supply for the Heartmate 2 is worn externally, connecting to the device via the system controller

**Fig. 2:**
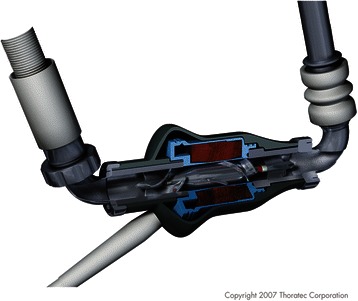
Cross section of Heartmate 2. Legend – The internal workings of the Heartmate 2 are shown in this schematic. The rotary pump is shown, as well attachments to the inflow and outflow tract

**Fig. 3:**
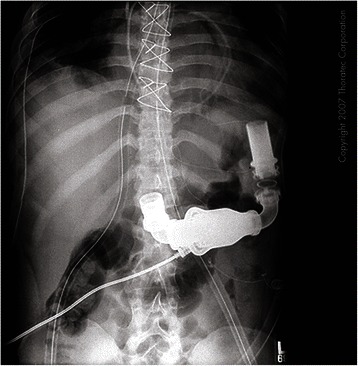
Chest X-ray showing radiographic appearance of Heartmate 2. Legend – This shows the radiographic appearance of the inflow and outflow tracts of the Heartmate 2 device. Note the power and control cable attached to the pump unit

The HeartMate II® (Thoratec Corp., Pleasanton, CA, USA) (Figs. 1, 2, 3) is a second-generation, axial flow, rotary pump approved for both bridge-to-transplant and destination therapy in the US, and is currently implanted in over 7000 patients [10]. Its speed is fixed and can only be adjusted by medical professionals. The external display of the device shows speed (RPM), power (W), pulsatility index, and flow (l/min) which is an estimated value based on power utilization. Typical pump speed ranges between 6000 and 15,000 RPM with pump power ranging from 6.8 to 15.5 W. Pulsatility index (PI) is a dimensionless value with a usual range of 3–4. The variation in pump flow (Q) is used to derive the PI: 10 × (Q_max_ – Q_min_)/Q_avg_. These values can be monitored on an external display, which can be useful clinically (i.e., flow as a surrogate for cardiac output) [10, 16].

**Fig. 4:**
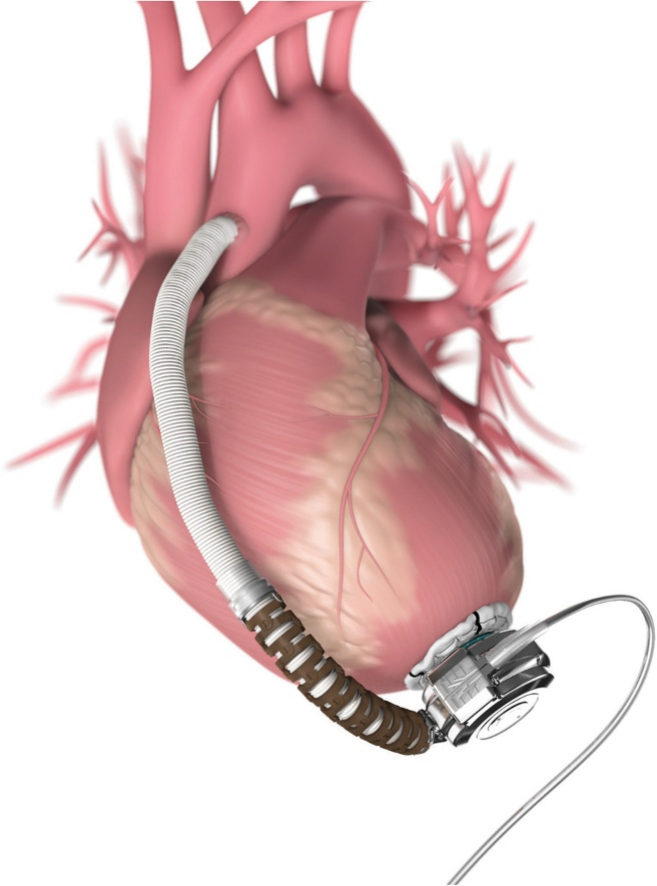
Heartware Device. Legend – HeartWare device implants directly into apex of left ventricle. Blood from LV passes through the device and into the left ventricle outflow tract

**Fig. 5:**
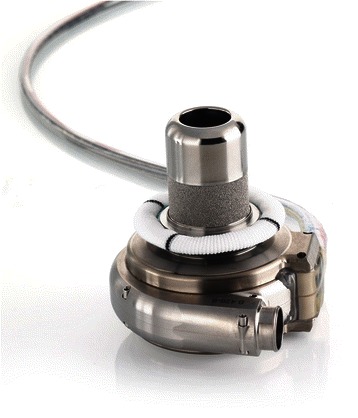
Heartware Device. Legend – The Heartware device implants directly into the left ventricle. The centrifugal pump design leads to a more compact device. The impeller is suspended using hydrodynamic forces to reduce the risk of thrombotic events

The HVAD™ (HeartWare Inc., Miami Lakes, FL, USA) (Figs. 4 and 5) is a third-generation centrifugal pump recently approved by the FDA for bridge-to-transplant in November of 2012. The most notable differences between the HeartMate II and the HVAD™ are its smaller size, centrifugal as opposed to axial pumping mechanism, and its lack of mechanical bearings. It utilizes hydrodynamic forces to suspend the impeller in an attempt to reduce the risk of mechanical failure, prolong pump life, and decrease the risk of thrombus formation [17]. Studies, however, have shown similar survival rates but increased incidence of stroke and gastrointestinal bleeding in patients with HVAD when compared to HeartMate II, though some reports have also shown similar rates of adverse events [2, 13]. Similar to the HeartMate II, the control screen displays pump speed, power, and an estimated pump flow, as well as graphical displays of pulsatility. Pump speed ranges between 1800 and 4000 RPMs and power ranges from 2.5 to 8.5 W [18]. The lower speed range of the HVAD™ is hypothesized to have less extreme hemodynamic effects and decreases the risk of creating sufficient negative pressure to cause “suction events” when a ventricular wall is drawn toward the inflow cannula causing complete or partial obstruction [13]. Overall, despite these differences, both the HeartMate II and HVAD™ can be managed with similar clinical principles.

#### LVAD Physiology

The physiologic changes associated with heart failure are complex and systemic. These patients clearly have reduced stroke volume and cardiac output associated with slow circulation times and decreased end-organ hypoperfusion. Compensation occurs via neurohormonal activation—high circulating catechol amines, natriuretic peptides, endothelin, and activation of the renin-angiotensin-aldosterone system—which results in ventricular remodeling. Hence, the mainstay of treatment is neurohormonal blockade [12]. The long-term effects of this malperfused and overly compensated state frequently result in hepatic, renal, or pulmonary insufficiency [19]. Continuous-flow LVAD implantation results in an off-loading of volume from the ventricle. This decompression results in decreased left ventricular work, reduces myocardial damage, improves chamber compliance, and favors “reverse remodeling” by improving left ventricular geometry [9]. The device improves arterial blood pressure and microcirculation, despite its non-pulsatile nature, which enhances end-organ perfusion and restores function [10]. These improved hemodynamics are also evidenced by reductions in the aforementioned neurohormonal response measured in the plasma [9]. Decompression of the LV also reduces pulmonary pressures and transpulmonary gradient, which is a factor that improves transplant candidacy [10]. Overall, LVAD implantation enhances end-organ and myocardial function, exercise tolerance, and overall quality of life [5, 9].

The device itself also depends upon several physiologic variables. The output of second- and third-generation devices is directly related to pump speed and inversely related to the pressure gradient across the pump [10, 16, 20]. These devices have continuously rotating impellers which continue to pump at a fixed speed regardless of their environment. For this reason, volume status and right ventricular (RV) function have a significant impact on LVAD flow and, therefore, cardiac output [16]. Despite a supported LV, patients may have coexisting right ventricular failure which may require inotropic support or pulmonary vasodilation to enhance RV stroke volume [19]. Afterload also has a large impact on LVAD function, as it directly impedes LVAD flow. Increases in systemic vascular resistance will have adverse effects on device output [16]. Pulsatility may be variable as the physiologic environment changes and is inversely related to how well the LVAD is off-loading volume from the native ventricle. If pulsatility increases significantly, it may be an indication of volume overload to the LV [10]. Overall, the goal is to maintain flow by ensuring adequate preload and RV contractility, while managing systemic vascular resistance to allow for optimal pump function.

Another important physiologic consideration after LVAD implantation is the coagulopathy associated with shear stress and nonpulsatile flow. Evidence suggests an acquired von-Willebrand Syndrome exists where a decreased amount of circulating high-molecular-weight von-Willebrand multimers are found, similar to that of severe aortic stenosis [10, 13, 21, 22]. Likewise, a coinciding platelet dysfunction occurs, which is more significant in nonpulsatile pumps than in their pulsatile predecessors [10]. These qualitative factors place patients at an increased risk of nonsurgical bleeding as evidenced by an increased incidence of gastrointestinal bleeding [20]. This risk is also increased due to the finding of arteriovenous malformations in the gut due to the nonpulsatile flow and decreased capillary pressure [13]. Thanks to this acquired coagulopathy, only mild levels of anticoagulation are required after implantation of second- and third-generation devices. Likewise, withholding anticoagulation perioperatively has been shown to be safe, which will be discussed further [1, 20, 23].

#### Preoperative evaluation

In addition to the standard components of a preoperative assessment, evaluation of a patient with an LVAD should include a few additional components. When possible, all LVAD patients undergoing non-cardiac surgery should have a multidisciplinary team of subspecialists coordinating their care. This should include not only the primary surgical and anesthesia teams, but also cardiac surgery, heart failure cardiologists, and dedicated VAD personnel [1, 7, 11, 20, 24, 25]. This may involve contacting the nearest tertiary care center for these resources, since the combined knowledge of these individual parties may be paramount to the successful care of these patients. It may also be necessary to contact the VAD manufacturer for emergency resources [26]. At times, the patient may also be best served by preoperative medical optimization guided by heart failure cardiologists [1, 24]. This multifaceted approach ensures optimal care of a complex patient throughout the perioperative period.

Next, baseline physical exam findings should be noted, with special concern to organ systems which may be affected by the baseline heart failure (renal, hepatic, or pulmonary) or those which may be affected by LVAD complications, such as a neurologic deficit secondary to a thromboembolic event. Assessment of laboratory values with attention to the same organ systems should be included [8, 26]. Many LVAD patients may also have implanted defibrillators or pacemakers, so an electrocardiogram should be reviewed and the consensus statement from the American Society of Anesthesiologists and the Heart Rhythm Society should be followed [16, 27]. A review of prior echocardiography findings, particularly transesophageal echocardiography (TEE), may be useful in order to compare baseline findings with an intraoperative evaluation, as hemodynamics can change throughout the procedure [20].

All modern LVAD patients will generally be maintained on anticoagulation, which will have to be managed preoperatively. Patients, therefore, will generally not be candidates for neuraxial anesthesia [8, 16, 26]. Both the HeartMate II and HVAD patients should have an INR of 2–3, aspirin 81 mg daily, and may include dipyridamole 75 mg three times daily or clopidogrel 75 mg daily [18, 28]. Recently, slightly lower INR goals of 1.5–2 have been recommended for the Heartmate II, and less stringent anticoagulation has recently been supported [2, 13, 29]. Warfarin should be discontinued at least 2–5 days preoperatively and patients should receive a heparin infusion which should be stopped the morning of the planned procedure [7, 16, 20, 30, 31]. For patients with a history of heparin-induced thrombocytopenia, both argatroban and bivalirudin have safely been utilized as well [32, 33]. As always, the risks of hemorrhage should be weighed against the risk of thromboembolic events, though the risk of bleeding is generally higher than the risk of thrombus with these devices, thanks to the aforementioned changes in platelet and von-Willebrand factor function [20, 22, 34]. In a case series of 20 patients with second- and third-generation devices, anticoagulation was transitioned to a heparin infusion (as above) without any thrombotic complications. They conclude that, if there are concerns for hemorrhage before or after surgery, anticoagulation can be safely held [1, 23]. Other case series have shown intraoperative erythrocyte transfusion requirements in 15–90 % of LVAD patients undergoing non-cardiac surgery, though the higher transfusion rates were found in a small series of 11 patients [1, 16, 34]. Overall, providers should transition anticoagulation appropriately, withhold it entirely if there are concerns for hemorrhage, and be prepared for transfusion when indicated. Patients awaiting transplantation should receive leukoreduced and irradiated blood products, though indications for transfusion are unchanged from patients without devices [35].

#### Hemodynamic monitoring

Intraoperative monitoring of LVAD patients presents unique challenges due to their nonpulsatile nature. Standard ASA monitors, such as pulse oximetry and noninvasive blood pressure monitoring, rely on pulsatility and oscillations, respectively. Cerebral tissue oxygenation (SctO2) measurements have been used successfully as a surrogate for pulse oximetry in these patients [11, 16, 26]. The monitor should be placed pre-induction while the patient is awake, and efforts should be directed at maintaining these baseline values throughout the procedure [20]. Serial arterial blood gas measurements are another alternative, though this approach is invasive and lacks the advantage of real-time data that cerebral oximetry provides [16]. Given concerns for infection, invasive monitoring should be avoided whenever possible. Pulmonary artery catheters are generally not required. Pump flow (an indicator of cardiac output) is displayed on the LVAD screen; therefore, pulmonary artery (PA) catheters provide little additional information. The exception to this may be patients with significant pulmonary hypertension at risk for right ventricular failure [8, 16, 20, 30, 34]. Noninvasive blood pressure devices may detect mean arterial pressure, but an arterial line should be placed (likely requiring ultrasound guidance) if hemodynamic fluctuations are expected [7, 8, 11, 16, 20, 24, 26]. In published case series, the use of arterial lines varies widely from 0 to 100 %, though they are generally indicated for most patients undergoing general anesthesia [1]. Central venous catheters may also be indicated if significant fluid shifts are expected [7, 8, 11, 20]. The monitor of choice, however, is the use of transesophageal echocardiography [8, 20, 30]. TEE allows full evaluation of important elements that effect LVAD function, such as volume status, RV function, inflow cannula position, and LV decompression [16, 26]. One final monitor to consider is the use of processed EEG, given the fact that hypertension and tachycardia may not be a reliable indicator of inadequate anesthetic depth in LVAD patients [16].

#### Intraoperative management

After preoperative optimization and coordination of a multidisciplinary team, when patient condition permits, the patient can be taken to the operating room. Patients should be transported to the operating room in coordination with VAD personnel who have knowledge of VAD intricacies, such as connecting the portable power supply, control console and changing VAD settings if necessary. Once in the operating room, the device should be attached to a secure power supply for the duration of the procedure [1, 7–9, 11, 20, 30]. Hemodynamic monitors should be placed as discussed above Table 1. Additionally, external defibrillator pads should be applied [16, 26]. Bipolar cautery should be utilized whenever possible, and the grounding pad should be placed away from the device to limit electromagnetic interference [4, 7–9]. Induction of anesthesia, when appropriate, may then proceed.

**Table 1:** Perioperative approach to LVAD patients undergoing non-cardiac surgery

• Preoperative
o Multidisciplinary team identified (primary surgical and anesthesia teams, cardiac surgery, heart failure cardiologist, VAD personnel)
o Preoperative medical optimization when possible or necessary
o Physical examination focused on the sequelae of heart failure
o Baseline EKG, echocardiogram, and laboratory values
o Manage pacemaker/AICD settings when indicated
o Hold, bridge, or reverse anticoagulation when indicated
• Intraoperative
o Standard ASA monitors
o Cerebral tissue oxygenation, processed EEG, arterial line with ultrasound guidance, central venous catheter if fluid shifts are expected, PA catheter only if severe pulmonary hypertension, TEE available
o Monitor VAD control console
o External defibrillator pads in place
o Optimize preload, support RV function, avoid increased in afterload
o Gradual peritoneal insufflations and position changes
• Postoperative
o Standard PACU care unless ICU is otherwise indicated
o Extubation criteria are unchanged
o Avoid hypoventilation, optimize oxygenation
o Resume heparin infusion when post-op bleeding risk is acceptable

The presence of an LVAD does not preclude the use of anesthetic drugs or techniques that would otherwise be acceptable for these patients. Standard induction agents and balanced anesthetic techniques have been used safely [7, 8, 11, 16, 20, 30]. Generally, however, LVAD patients would not be candidates for neuraxial anesthesia given their anticoagulation status. Standard laryngoscopy and intubation is acceptable for patients with second- and third-generation devices implanted in the pericardial space; however, patients with first-generation devices implanted in the peritoneal space should be considered an aspiration risk and induced using a rapid sequence technique [8, 16, 20, 26, 30].

Goals of care for LVAD patients undergoing non-cardiac surgery should be directed at maintaining forward flow and adequate perfusion. Three main factors that affect LVAD flow are preload, RV function, and afterload. First, optimizing preload includes ensuring adequate volume status without overloading the right ventricle [7, 9, 11]. Acute volume overload may precipitate isolated RV failure in 20–30 % of LVAD patients [30]. However, a careful assessment of volume status should be completed as certain patients may benefit from preoperative volume loading, depending on the clinical scenario [7]. Maintaining spontaneous ventilation, when possible, may augment venous return as well [8, 16, 30]. When positive pressure ventilation is necessary, adequate oxygenation and ventilation should be balanced with minimizing intrathoracic pressure, which adversely affects preload [16].

The right ventricle is the primary means of LVAD filling; therefore, maintaining RV function is imperative [26]. Negative inotropes should be used with caution [7]. Any decreases in oxygenation, ventilation or high levels of positive end-expiratory pressure will increase pulmonary vascular resistance and thus adversely affect RV function [7, 9, 11, 26]. RV strain will then worsen hypoxia and respiratory acidosis and further exacerbate RV failure and decrease VAD flow. With appropriate safeguards, even one-lung ventilation has been performed safely in an LVAD-dependent patient without changes in oxygenation, ventilation, or hemodynamics [16]. Finally, care should be taken to manage systemic vascular resistance as afterload directly impedes LVAD flow. Marked increases in systemic vascular resistance should be avoided [11]. Sympathetic discharge from laryngoscopy or surgical stimulation may cause an overt rise in afterload; for this reason, adequate anesthetic depth should be ensured to avoid any abrupt change in systemic vascular resistance [7, 9]. Not only does decreased VAD flow impair peripheral perfusion, but it may also promote stasis of blood in the device and increase the risk of thromboembolic events [7, 11].

Keeping in mind the LVAD’s preload dependence and afterload sensitivity is key to managing changes in hemodynamics [7, 20, 34]. Though displayed VAD flow is an estimate based on power utilization, these values should be trended carefully throughout the procedure [16]. Changes in VAD flow should be combined with other available information, such as operative conditions, arterial pressure, central venous pressure, and TEE findings to diagnose the etiology of decreased flow. Generally, decreases in pump flow should first be treated with a fluid challenge. Hypovolemia should be avoided and intraoperative losses should be replaced aggressively [11, 26]. Second line treatment should include inotropic support for the right ventricle [7, 20]. For example, decreased LVAD flow and a continuously rising central venous pressure may be indicative of RV failure. In this case, appropriate management would include supporting the right ventricle with inotropes and pulmonary vasodilators, as opposed to a fluid bolus [7, 9, 11, 16, 26]. If, however, hypotension is accompanied by increases in LVAD flow, this may be due to decreased systemic vascular resistance. In this case, vasopressors should be titrated carefully to balance adequate perfusion pressure with forward flow [16]. Low-dose vasopressin (<2.4 U/h) may be the vasopressor of choice due to its minimal effect on pulmonary vascular resistance [11, 26]. Cerebral tissue oxygenation should also be monitored carefully throughout the procedure. Significant decreases from baseline should first be treated with increased inspired oxygen. If the decreased SctO2 is accompanied by decreases in LVAD flow, it should be treated as described above with first a fluid challenge, then inotropic support [20].

Managing arrhythmia may present a unique challenge in LVAD-dependent patients. Many patients may have implanted defibrillators or pacemakers which may require reprogramming prior to the procedure [16, 27]. External defibrillator pads should be in place [16, 26]. Patients should be kept in normal sinus rhythm when possible. Arrhythmia may impair the unassisted ventricle and decrease inflow to the LVAD [20]. Standard Advanced Cardiovascular Life Support Guidelines should be followed; however, external chest compressions should be avoided during cardiac arrest [26, 36]. Compressions risk cannula dislodgement, which may result in life-threatening hemorrhage [7, 8, 16].

Intraoperative events that affect preload and afterload should be completed cautiously. Position changes should be performed gradually and extremes in position should be avoided. Preoperative volume loading has been shown to stabilize hemodynamics when positions are required that decrease preload, such as lateral decubitus or reverse Trendelenburg [7, 8, 16, 26, 30]. Likewise, steep Trendelenburg may increase venous return, risking RV strain. Peritoneal insufflation for laparoscopic surgery also increases afterload and has detrimental effects on preload. Insufflation should utilize minimum pressures and be increased in a gradual, step-wise fashion [7, 16, 20, 26]. However, standard insufflation pressures have been utilized safely for laparoscopic surgery [7, 20, 37].

Managing device complications may present a challenge that requires a multidisciplinary team to diagnose and treat [38]. Motor failure is an extremely rare complication that presents with symptoms of worsening heart failure, increased pulsatility, and decreased arterial pressure. Patients require urgent consultation with cardiac surgery for possible replacement. A more common device issue is obstruction of the inflow or outflow cannula. This may be caused by thrombus, a kinked cannula, or a “suction event”. Obstruction presents as decreased pump flow, decreased arterial pressure, and increases or decreases in pump power. TEE can be extremely valuable in diagnosing the cause of obstruction. A suction event occurs when the LV is under filled due to hypovolemia or RV failure. This causes the intraventricular septum or free wall of the ventricle to collapse against and obstruct the inflow cannula. This presents as with the signs of obstruction and may include tachyarrhythmia as well. If this is suspected, it should be aggressively treated with a fluid challenge. Decreasing the pump speed temporarily may also allow for improved LV filling, though this requires close coordination with the VAD personnel [34].

#### Postoperative management

In general, the postoperative care of LVAD-dependent patients undergoing non-cardiac surgery is largely uneventful. Patients should be extubated when they meet standard criteria [20, 24, 30]. Special care should be taken to ensure acceptable oxygenation and ventilation to avoid changes in pulmonary vascular resistance [16]. Most patients can be recovered in the standard post-anesthesia care unit, unless intensive care is otherwise indicated [1]. A major concern in the postoperative course will be the resumption of anticoagulation. Patients who are both anticoagulated and on antiplatelet therapy are at risk for hemorrhagic postoperative complications [34]. However, heparin infusion should be resumed when the risk of postoperative bleeding is acceptable [4]. Case series have reported resuming heparin infusions immediately following surgery or up to 26 h later [34, 37]. There are also reports of foregoing heparin infusion entirely and transitioning directly to oral anticoagulants without increased risk of thromboembolism [23]. Oral anticoagulation can be resumed when bleeding from surgical drains ceases, though the heparin infusion should be continued until the patient reaches their goal INR [24, 34]. Aspirin management varies widely in previously reported cases, though most report the resumption of aspirin one week postoperatively [34]. Again, second- and third-generation devices have a low thromboembolic risk so the risk of perioperative hemorrhage may be greater than the risk of thrombus formation; therefore, adequate hemostasis should be ensured prior to the resumption of full anticoagulation [20, 23].

## Conclusions

There is an increasing frequency of LVAD-dependent patients presenting for non-cardiac surgery. These trends will likely increase due to advances in cardiac care and the consequent increase in life expectancy and prevalence of end-stage cardiac disease. The general anesthesiologist needs to be prepared to evaluate these patients and mitigate cardiac risk in the perioperative period. The unique challenges of this patient population can best be addressed by a thorough understanding of LVAD physiology and a multidisciplinary approach to care.

## Abbreviations

FDA: Food and drug administration
LV: Left ventricle
LVAD: Left ventricular assist device
NHYA: New York Heart Association
PA: Pulmonary artery
PI: Pulsatility index
RV: Right ventricle
TEE: Transesophageal echocardiography
VAD: Ventricular assist device
